# Using Underwater Pulse Oximetry in Freediving to Extreme Depths to Study Risk of Hypoxic Blackout and Diving Response Phases

**DOI:** 10.3389/fphys.2021.651128

**Published:** 2021-04-01

**Authors:** Eric Mulder, Arne Sieber, Erika Schagatay

**Affiliations:** ^1^Environmental Physiology Group, Department of Health Sciences, Mid Sweden University, Östersund, Sweden; ^2^Swedish Winter Sports Research Centre, Mid Sweden University, Östersund, Sweden

**Keywords:** breath-hold diving, apnea, arterial oxygen saturation, syncope, heart rate, bradycardia, exercise, oxygen conservation

## Abstract

Deep freediving exposes humans to hypoxia and dramatic changes in pressure. The effect of depth on gas exchange may enhance risk of hypoxic blackout (BO) during the last part of the ascent. Our aim was to investigate arterial oxygen saturation (SpO_2_) and heart rate (HR) in shallow and deep freedives, central variables, which have rarely been studied underwater in deep freediving. Four male elite competitive freedivers volunteered to wear a newly developed underwater pulse oximeter for continuous monitoring of SpO_2_ and HR during self-initiated training in the sea. Two probes were placed on the temples, connected to a recording unit on the back of the freediver. Divers performed one “shallow” and one “deep” constant weight dive with fins. Plethysmograms were recorded at 30 Hz, and SpO_2_ and HR were extracted. Mean ± SD depth of shallow dives was 19 ± 3 m, and 73 ± 12 m for deep dives. Duration was 82 ± 36 s in shallow and 150 ± 27 s in deep dives. All divers desaturated more during deeper dives (nadir 55 ± 10%) compared to shallow dives (nadir 80 ± 22%) with a lowest SpO_2_ of 44% in one deep dive. HR showed a “diving response,” with similar lowest HR of 42 bpm in shallow and deep dives; the lowest value (28 bpm) was observed in one shallow dive. HR increased before dives, followed by a decline, and upon resurfacing a peak after which HR normalized. During deep dives, HR was influenced by the level of exertion across different diving phases; after an initial drop, a second HR decline occurred during the passive “free fall” phase. The underwater pulse oximeter allowed successful SpO_2_ and HR monitoring in freedives to 82 m depth – deeper than ever recorded before. Divers’ enhanced desaturation during deep dives was likely related to increased exertion and extended duration, but the rapid extreme desaturation to below 50% near surfacing could result from the diminishing pressure, in line with the hypothesis that risk of hypoxic BO may increase during ascent. Recordings also indicated that the diving response is not powerful enough to fully override the exercise-induced tachycardia during active swimming. Pulse oximetry monitoring of essential variables underwater may be an important step to increase freediving safety.

## Introduction

Freediving is increasing in popularity both as a recreational and competitive sport (Divers Alert Network, 2017), where all activities are performed on one breath of air. While most freediving is done in the depth range down to 20 m, deep freediving is an increasingly popular extreme sport where elite divers reach far beyond that depth, exposing themselves not only to progressive hypoxia but also to dramatic changes in pressure. These changes have several physiological effects, both by direct compression of air filled cavities (Boyles law) and on gas exchange, as partial pressure of oxygen changes proportionally with ambient pressure (Dalton’s law). In competition freediving; “Apnea” there are four disciplines with the aim to reach the greatest possible depth on one breath, and to return back to the surface with one’s own muscular effort, and to date competitive freedivers have reached 130 m using a monofin and 102 m swimming without fins (International Association for the Development of Apnea, 2021). A typical deep freedive is characterized by four different phases, with different physiological demands, as buoyancy may help or counteract the swimming efforts (Schagatay, 2011).

The most obvious limitation to deep freediving is the freediver’s maximal breath-holding capacity, as the diver cannot immediately return to the surface once depth has been reached. Therefore, although all freedivers are exposed to risk of hypoxic syncope (Craig, 1961; Lin et al., 1974), which is called “blackout” (BO) by the divers, its consequences may be more severe in deep diving. The effect of pressure on gas exchange may in addition expose deep divers to increased risk of blackout resulting from the air re-expansion during ascent, which can dramatically reduce arterial oxygen saturation (SpO2), resulting in a specific case of blackout often called “shallow water blackout” (Lanphier and Rahn, 1963; reviewed in Schagatay, 2011).

While during the descent, the increase in hydrostatic pressure leads to an increment in alveolar partial pressure of oxygen, which may result in a temporary state of hyperoxia (Linér et al., 1993), during ascent the pressure diminishes with a concomitant decrease in alveolar oxygen pressure, which may invert the flow of oxygen at the alveolar level. This situation may cause the SpO_2_ to drop very quickly, and cause an enhanced risk of hypoxic blackout near the surface (Linér et al., 1993; Ferretti, 2001). Although this situation is considered to be a major cause of deaths in freediving, continuous measurements of SpO_2_ during deep dives are currently lacking. This is mainly because physiologists have up to now been highly dependent on access to the studied person to make direct measurements of physiological variables on the body using a range of methods and equipment. There exist few technological devices allowing recording of physiological values underwater, and there is no possibility to follow a diver underwater to extreme depths using SCUBA gear, as the freedivers move so rapidly vertically across the water column, that this could cause decompression illness in the SCUBA diver. Most research in freediving physiology to date is therefore based on laboratory studies (Lin et al., 1974; Schagatay and Andersson, 1998; Bakovic et al., 2003; Lemaitre et al., 2007) or at the water surface (Marongiu et al., 2015; Fernández et al., 2019). Only two previous studies have used pulse oximetry during freediving to shallow depth (Stanek et al., 1993; Kuch et al., 2010), and another few have measured heart rate (HR) during deep dives (Schagatay, 2011; Lemaitre et al., 2013).

In order to further investigate the human physiological adaptations during actual freediving to great depth, new devices must be developed for recording central variables remotely. But which variables are most central to determine a freediver’s physiological performance limits and exposure to risks? While several factors determine the diver’s ultimate depth limits (Schagatay, 2011), maximal diving duration is set by the diver’s total gas storage capacity, the lowest tolerable oxygen levels, and metabolic rate, i.e., the time it takes for the diver to use up the available oxygen stores (Schagatay, 2009). Individual oxygen storage capacity can be measured before the dive in dry conditions, and a test determining the individual tolerance to hypoxia could also be done in a non-immersed diver. However, SpO_2_ measured during the dive would reveal remaining available oxygen stores and therefore be central to predicting diving duration, together with metabolic rate. Metabolic rate relates to physical activity, but can be transiently limited by the cardiovascular “diving response,” which conserves oxygen by reducing peripheral circulation and myocardial oxygen consumption, leading to enhanced SpO_2_ and longer breath hold duration (Andersson and Schagatay, 1998; Schagatay and Andersson, 1998). Therefore, most central for determining metabolic rate and oxygen metabolism during freediving is monitoring of HR and SpO_2_ during the dive, two variables that can be simultaneously measured *via* pulse oximetry.

We therefore developed a pressure- and water-resistant pulse oximeter. Our immediate study aim was to monitor HR and SpO_2_ during freedives to great depth in order to (1) determine if good quality data could be collected at 80 m depth, and (2) to establish if HR and SpO_2_ patterns differ between shallow and deep dives, and whether HR differed between the different diving phases of deep dives. The long-term goal is to use this monitor to enhance the understanding of the physiological events during deep freediving and to be able to predict safe diving duration and thereby promote freediving safety.

## Materials and Methods

### Participants

Four healthy male elite competitive freedivers (mean ± SD age 38 ± 12 years, height 181 ± 10 cm and weight 76 ± 12 kg) volunteered to be part of this study during their regular freediving training. They all had a minimum of 5 years of freediving experience, and qualified in category 5 in the 5-level categorization system used for freedivers with respect to their training/experience (Schagatay and Andersson, 1998). The mean personal best competition performance in constant weight (CWT) deep diving with fins was 91 ± 10 m.

The freedivers were well acquainted with and used the safety procedures established in competitions, e.g., to be accompanied during the last 20 m toward the surface by a safety diver. All participants were given verbal information regarding the purpose of the study and signed an informed consent prior to engaging in their pre-diving routines. The study protocol had been approved by the Regional Committee for Medical and Health Research Ethics in Umeå, Sweden, (Dnr 2019-05147) and tests were conducted in accordance with the 2004 Declaration of Helsinki.

### Diving Procedures and Data Recording

When the freedivers had finished their pre-dive routines, they were equipped with a prototype pulse oximeter. The prototype pulse oximeter was evaluated prior to the current study and results will be presented elsewhere. Briefly, the prototype consisted of a ruggedized universal datalogger platform based on a ST Microelectronics STM32L452 microprocessor (STMicroelectronics International N.V. Amsterdam, The Netherlands), with a 32 bit ARM Cortex M4 core, while a 32 GB micro-SD card was integrated for data storage.

The prototype was equipped with two SpO_2_ probes based on the MAXIM MAX30102 chip (max 50 Hz sampling rate) and an ambient pressure sensor. The MAX30102 (Maxim Integrated, CA, United States) sensor frontend recorded plethysmograms and includes red and infra-red emitters, diode drivers, photodiode, photodiode amplifier, analog to digital converter, and controller. Distance between light/infra-red emitter and photodiodes was 3 mm. A graphical user interface was developed in Labview (National Instruments Corporation, Austin, United States), which was used to show all parameters in real time and display the recordings.

Sample algorithms for SpO_2_ calculations in low noise environments were supplied by the manufacturer (Maxim Integrated, CA, United States); however, artifacts of different kinds may lead to incorrect SpO_2_ calculations. The algorithm was therefore optimized and included an auto-correlation algorithm to filter motion artifacts. More specifically, the SpO_2_ algorithm was based on calculation of the Root Mean Square (RMS) value of alternating current (AC) and direct current (DC) of the red and infrared channel as described by the manufacturer (Application note 6845, Maxim Integrated, CA, United States). An improved version of this algorithm was also performing a correlation between infrared and red signal to calculate a measure of signal quality. In an undisturbed signal, infrared and red signals correlated well, and in case of bad correlation, the calculated values were discarded. The previous evaluation of the prototype showed that SpO_2_ and HR seemed in good agreement with those measured of two conventional finger pulse oximeters during normoxic and hypoxic breathing, as well as during body immersed static apnea.

**Figure 1:**
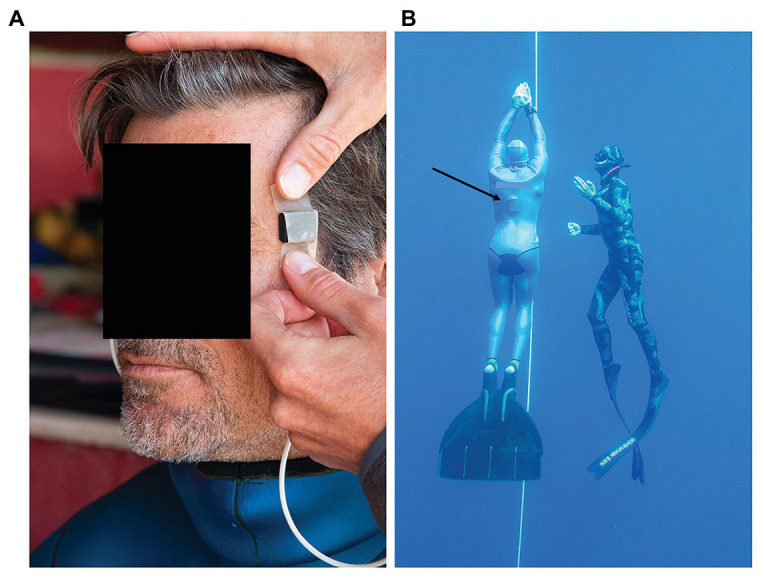
**(A)** Sensor heads of the pulse oximeter probes were applied to the temple and fixated with medical tape, and thereafter by pressure from the hood of the wetsuit, **(B)** freedivers performed their training dives from a buoy and dove to their predetermined depth along a vertical line, and were accompanied up by a safety diver. Black arrow points at the recording unit of the pulse oximeter located underneath the wetsuit.

The sensor heads of the two probes were applied to the temples, and fixated with medical tape as well as by the pressure from the hood (Figure 1A). If the hood was loose, an extra head band or swim cap was used to apply further fixation. The quality of the plethysmograms signal was inspected and, if considered adequate, the data logger was inserted under the wetsuit, and placed on the back of the freediver (Figure 1B). This ensured that no cables were outside of the wetsuit, thus not compromising the safety of the freediver.

To enable visual inspection of the quality of the plethysmograms prior to the diving session, the recording unit was connected to a computer through an optical fiber output cable, which allowed real-time transmission of the data. The cable was then removed and the recording was manually started. The prototype pulse oximeter registered plethysmograms at 30 Hz for continuous SpO_2_, HR, and depth recordings. The mean ambient air temperature was 26°C and sea surface temperature was 22°C.

The freedivers entered the water and performed their planned training dives from a buoy starting the dives on their own volition (Figure 1B). The divers did not follow an imposed diving protocol; however, they all performed at least one shallower freedive to approximately 20 m depth, and one deep freedive to at least 50 m. Dives were prepared according to each divers’ own preparatory routines, the last part of which involved lung packing to maximize lung volume (Örnhagen et al., 1998). After surfacing the divers used hook breathing for facilitated recovery (Fernández et al., 2019). The recording was manually stopped and the equipment was removed once the divers had completed their training session and exited the water.

### Data Analysis

Arterial oxygen saturation and HR were extracted from the plethysmograms at a window size of 6-s, and analyzed internally every second. Since the pulse oximeter included two probes, the mean was calculated from the results of both sensors to obtain one single value for SpO_2_ and HR for each second. *Dropout rate* was presented and refers to interruptions in continuous SpO_2_ and/or HR data due to down time or machine-probe unit nonfunction, and calculated as the percentage of time when SpO_2_ and/or HR data were not provided, as defined by Barker and Shah (1997). The resulting time series for each dive was then smoothened using a 5-s moving median and subsequently a 5-s moving average.

In order to follow the continuous events, a graph of HR and SpO_2_ was drawn based on the three dives with the longest durations for shallow and deep dives, respectively. The exclusion of the two dives of shortest duration was done to provide a longer period of mean values. The presented data for the three included dives were synchronized at two points (1) at the dive initiation, and (2) at surfacing at the end of the dive, both marked as “0” on the time axis, with the middle part of the longer dives omitted.

The nadir SpO_2_ and HR values for each dive and the time of their occurrence were noted. Baseline values of SpO_2_ and HR were recorded during rest at the surface between 5 and 15 min before the first dive. The maximal arterial oxygen desaturation was calculated as the percent change from baseline for the lowest 1 s SpO_2_ value occurring between the start of the dive and 30 s after surfacing, to account for the circulatory delay. The maximal magnitude of diving bradycardia was evaluated as the percent change between the baseline HR and the lowest 1 s HR value recorded during the dive. For each diver, the different diving phases were identified through the depth profile and change of descent speed, and the mean HR calculated for each phase.

### Statistics

Calculated data are presented as mean ± SD for *n* = 4, unless otherwise stated. As subjects served as their own controls, paired Student’s *t*-test for selected data points, with Bonferroni correction for repeated tests, was used to compare diving phases and maximal responses between shallow and deep dives. Significance was accepted at *p* < 0.05. The sample size of the current study was small and responses were in part determined by direct inspection of individual and mean data graphs.

## Results

### Data Quality

There were periods of apparent motion artifacts in the recordings from one or both sensors, resulting in missing data points. Loss of data most often occurred at the start of the dive, when the diver performed lung packing prior to the dive, and during the first phase of the descent, when middle ear equalization needs to be done frequently. During the last phase of the ascent near the end of dives, loss of data again occurred more often, most likely linked to increased frequency and magnitude of involuntary breathing movements, and possibly as a result of SpO_2_ being extremely low, which makes it harder for the reflective probe to detect oxygen saturation. The dropout rate of 1 s values for all trials was 34% and after smoothing it was 0.5%.

### Diving Patterns and Maximal Responses

Depth of the shallow dives was 19 ± 3 m, while it was 73 ± 12 m for the deep dives (*p* = 0.003; Table 1). The deep dives performed were to 81 ± 10% of the diver’s personal best depth competition performance in the discipline CWT. Duration of the shallow dives was 82 ± 36 s and 150 ± 27 s for the deep dives (NS).

**Table 1:** Individual and mean (SD) baseline reference values of SpO_2_ and HR during a period of immersed rest before dives, followed by depths, durations, and nadir SpO_2_ (%) and HR (bpm) values during deep and shallow dives; time when nadir occurs is noted as seconds from start of the dive, as well as the maximal reduction from pre-dive SpO_2_/HR reference values (%).

					Shallow				
Diver	Pre SpO_2_	Depth	Duration	SpO_2_ nadir	SpO_2_ nadir (s)	SpO_2_ red (%)	HR nadir	HR nadir (s)	HR red (%)
1	99.6	19	97	97	116	3	28	33	67
2	97.3	15	32	89	41	8	46	29	23
3	99.2	23	80	48	89	52	59	49	37
4	98.3	20	117	85	136	13	36	97	55
Mean	**98.6**	**19.1**	**81.5**	**80.0**	**95.5**	**18.9**	**42.1**	**52.0**	**45.6**
SD	1.0	3.1	36.3	21.9	41.1	22.3	13.4	31.2	19.1
	*p* =	0.003	0.117	0.040	0.144	0.041	0.978	0.635	0.987
**Diver**	**Pre HR**				**Deep**				
1	84.0	72	149	68	157	32	34	31	59
2	60.0	82	183	54	178	45	38	122	37
3	93.9	82	148	44	144	55	48	61	49
4	78.8	57	118	54	133	45	49	54	38
Mean	**79.2**	**73.3**	**149.5**	**55.0**	**153.0**	**44.3**	**42.3**	**67.0**	**45.7**
SD	1.0	11.8	26.6	9.7	19.3	9.6	7.2	38.8	10.6

*p*-values are shown for comparisons between deep and shallow dives.

All divers desaturated more during the deeper dives with a mean nadir SpO_2_ of 55% compared to the shallow dives, when SpO_2_ dropped to a mean nadir SpO_2_ of 80% (*p* = 0.040; Table 1). The lowest individual SpO_2_ observed was 44% after a deep dive, and this diver had a low value, 48%, also after the shallower dive. The nadir SpO_2_ was observed near or just after the end of the deep dives, and with some more delay after surfacing from the shallower dives (Table 1).

The maximal heart rate reduction, characterizing the diving response, was the same in shallower (42 bpm) and deep dives (42 bpm; NS) and in both cases represented a mean HR reduction of 46% from the pre-dive level (Table 1). The lowest HR observed was 28 bpm, and the time point of occurrence of the lowest HR was similar at 52 s for shallow and 67 s for deep dives (NS; Table 1).

### SpO_2_ and HR Patterns

**Figure 2:**
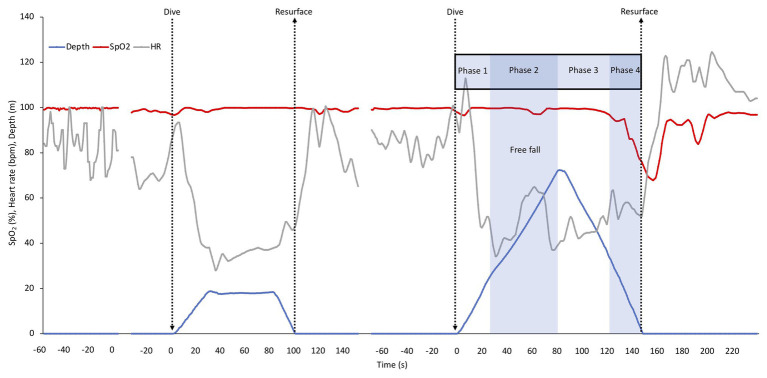
Individual continuous recordings from Diver 1 of arterial oxygen saturation (SpO_2_), heart rate (HR), and depth during immersion before starting to dive, during the shallow and the deep dive followed by a recovery period. The interruptions represent about 5 min of cut out data. The typical phases of a deep dive are indicated. In a deep dive, i.e., when lungs are compressed below residual volume which for most individuals occurs at greater depth than 30 m, four distinct phases can be identified; (1) a phase of active swimming against positive buoyancy, (2) a passive “free fall” phase when the diver can relax and fall as a result of negative buoyancy, which is after the turn followed by (3) a phase of intense swimming, when the diver actively swims upward against negative buoyancy and (4) the last phase of the dive, when swimming is aided by positive buoyancy.

Individual recording of data (after smoothing) of the shallow and deep dive for diver one is shown in Figure 2, with the phases of the deep dive marked. In the shallow dive, this diver stayed at 20 m for a longer period, doing a training “hang,” while in the deep dive the bottom turn only lasted for a few seconds (Figure 2). The individual dive data shown represented the least interrupted data of the four deep dives. The SpO_2_ in this diver was nearly unaffected in the shallow dive, while it dropped toward the end of the deep dive, with nadir SpO_2_ occurring just after surfacing due to the circulatory delay (Figure 2). HR showed a diving bradycardia of similar magnitude in both dives, but with a more variated HR in the deeper dive. The nadir HR of approximately 30 bpm occurred at about 30 s into both dives. After resurfacing, the HR increased above pre-dive levels, and returned to baseline after approximately 1 min, a recovery increase which was greater after the deep dive (Figure 2). In the deep dive of this diver, HR suddenly increased before reaching the target depth, which could be a result of intense equalization.

In all divers HR varied more in the deep dives. For three of the four divers SpO_2_ traces remained stable and high during the descent, and dropped during ascent, while one diver started to desaturate already during the last part of the descent in both the shallow and the deep dive.

**Figure 3:**
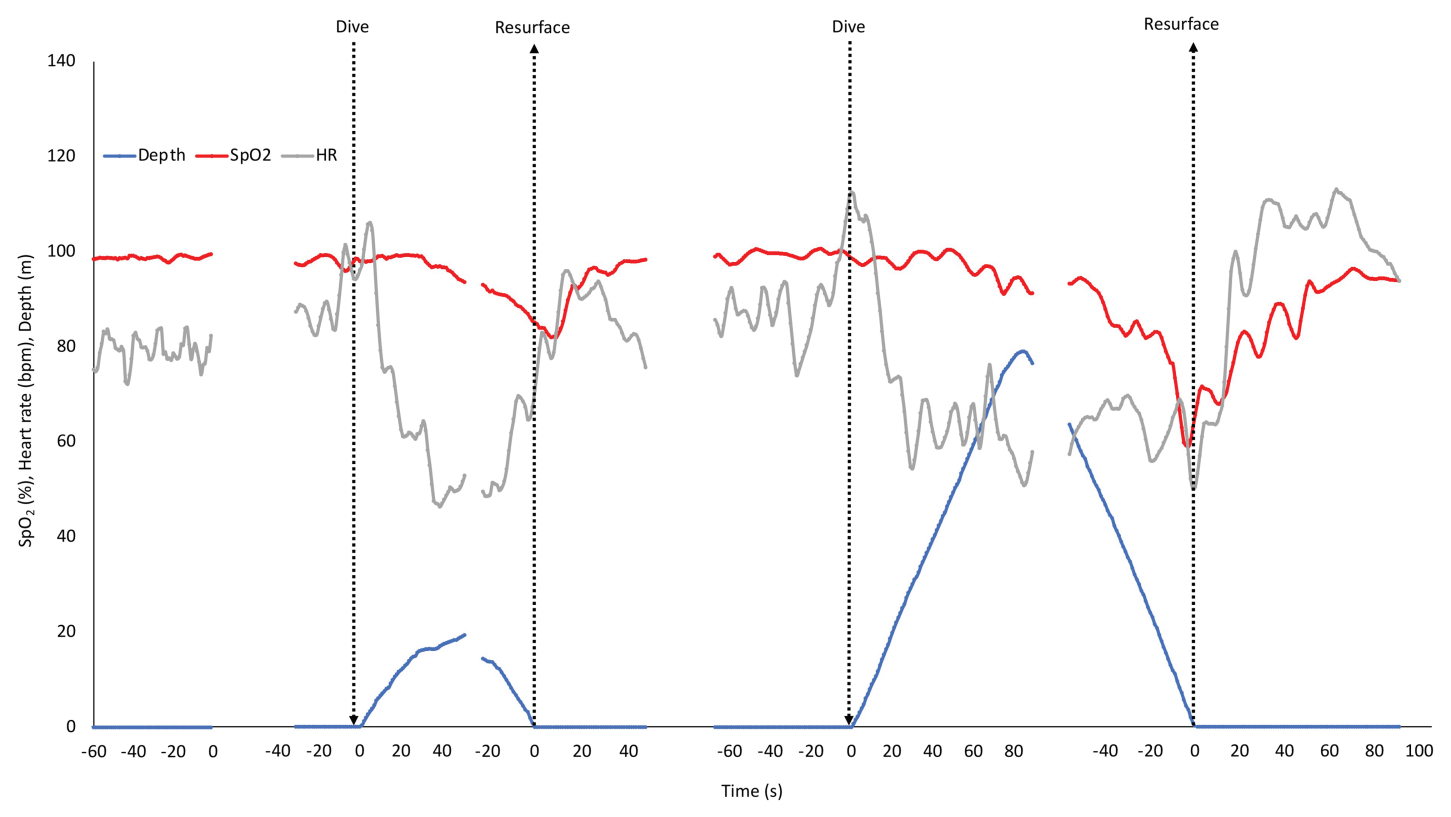
Mean depth, SpO_2_, and HR response pattern during rest before dives and in three of the four shallow and deep dives. SD has been left out for clarity. The shallow and deep dives with the shortest durations were not included to allow longer means. Arrows indicate start and end of dives, and also marks the two points, where dives were aligned for calculations. Gaps represent ommitted parts in the middle of the dives with the longest duration.

The mean HR and SpO_2_ response patterns for the shallow and deep dives for three of the divers (with exclusion of the two dives with shortest duration) is shown in Figure 3. The SpO_2_ patterns revealed a moderate desaturation toward the end of the shallow dive, and a more pronounced desaturation after the deeper dives (Figure 3). The rate of decline in SpO_2_ appeared to be increased in the deeper dive.

Heart rate responses were similar in shallow and deep dives. An initial increase at the start of the dive was followed by a “diving response” with a progressive drop in HR during the descent with nadir reached at or near the bottom. During ascent HR rose again, but dropped somewhat when divers were approaching the surface, and increased again during recovery after which it normalized (Figure 3).

### Diving Phases

**Figure 4:**
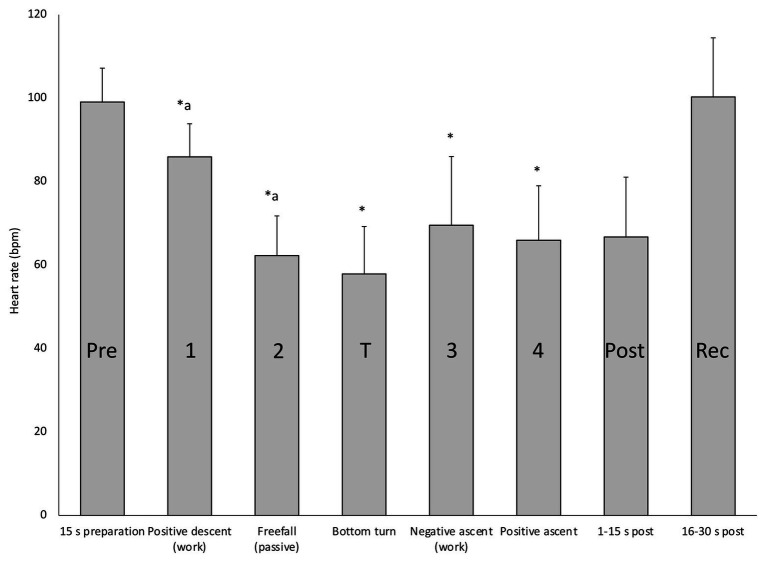
Mean (SD) HR in each of the four deep diving phases, before (Pre) and at the turn of the dives, as well as immediately after surfacing (Post) and during recovery (Rec). ^∗^ indicates significant difference (*p* < 0.05) from the pre-dive value; “a” indicates difference from the previous phase.

When HR responses across all four deep dives were analyzed in detail, broken down to means for the separate phases related to the required energetics for swimming, the response patterns became more evident (Figure 4). During preparation before the dive, HR rose to above the pre-dive resting levels (Figure 4; Table 1). During positive descent (Phase 1) HR declined immediately from the pre-dive value, and declined further during the passive “free fall” (Phase 2), when divers stopped swimming, while at the bottom turn it remained on a similarly low level (Figure 4). When the diver started ascending by intense swimming (Phase 3) HR clearly increased but dropped again when positive buoyancy reduced the energetic demands (Phase 4). During the initial 15 s after surfacing, when SpO_2_ was still reduced, HR was low, but during the next 15 s it increased to the pre-dive level (Figure 4).

## Discussion

The newly developed water and pressure-proof pulse oximeter allowed successful monitoring of both SpO_2_ and HR in freedivers down to 82 m depth – to our knowledge the greatest depth where HR and SpO_2_ has been measured. There were short periods of data losses, but by using an averaging function, continuous data could be obtained for the main portion of the dives, revealing interesting response patterns of both SpO_2_ and HR.

### Arterial Oxygen Desaturation

While mean SpO_2_ was kept at pre-dive levels for the first third of the dives, this was followed by a decline in both shallow and deep dives. SpO_2_ declined more and apparently at a faster rate during the latter part of ascent from the deep dives. Divers’ enhanced desaturation during the deep dives was likely in part the result of an increased level of exertion and the extended dive duration. However, the increased rate of desaturation during ascent could be related to the rapidly diminishing pressure, with consequences for oxygen uptake (Muth et al., 2003). This situation could lead to a reduced or even reversed gas exchange during the ascent, when lung oxygen partial pressure may become lower than the corresponding pressure in the blood, leading to transfer of oxygen from the blood back to the lungs (Lanphier and Rahn, 1963). This observation is in line with the hypothesis that the risk of hypoxic blackout may increase during the ascent from deeper dives.

The extremely low SpO_2_ values observed at or near the end of the deep dives would be considered too low to support consciousness in non-divers (Lindholm and Lundgren, 2009). In experienced divers, apneic training has been suggested to have resulted in increased hypoxia tolerance, also at the neural level (Lindholm and Lundgren, 2006), a conclusion which is supported by our results. While three of our divers showed marginal to moderate desaturation in the shallower dives, in one diver, a shallow dive to 23 m resulted in a SpO_2_ of 48%, nearly as low as the 44% observed after the deep dive to 82 m lasting nearly twice as long. This illustrates that great individual differences in desaturation rate and most likely proneness to hypoxic syncope may exist, and also shows that other dive characteristics than depth and duration determine oxygen cost. In line with this, the diver had a fairly high HR especially in the shallow dive, reflecting more intense exertion or a less pronounced diving response suggesting a higher oxygen cost, which is supported by several previous studies done in the laboratory (Andersson et al., 2002, 2004; Lemaitre et al., 2007).

During recovery after the dives, “hook breathing”, a breathing maneuver developed by the divers to overcome hypoxia (Fernández et al., 2019), elevates SpO_2_, apparently in steps with some drop back between individual hook maneuvers. With the elevated HR reflecting a high cardiac output during the early part of recovery, transition time in the lungs could perhaps be so high that this may not allow full saturation to occur, however, further investigation needs to confirm this hypothesis. The fact that SpO_2_ patterns describe similar events as those observed in the laboratory support that such data can be efficiently and correctly collected on depth. This is further supported by the similarity in our observed HR response, compared to previous work.

### Heart Rate Responses During Diving Phases

The HR recordings clearly showed a “diving response” in both shallower and deep dives, in line with several studies in the lab and a few done in the field (Ferrigno et al., 1997; Andersson et al., 2002, 2004; Lemaitre et al., 2008, 2013; Schagatay, 2011). HR traces for all divers and dives had similar features: a rise in HR during preparation with lung packing prior to the dive, followed by a decline in HR during the descent phase and, upon resurfacing, a peak in HR during hook breathing and recovery, after which HR normalized. While response patterns were similar, the levels of bradycardia differed between individuals.

During the deeper dives, HR was more irregular, which was likely a result of the larger variations in muscular work and pressure. Four clear phases of the deep dives could be identified which involved different HR levels, showing that different energetic demands during these phases likely affected HR despite an active diving response. The descent in the deep dives had two distinct phases, with a second drop in HR during free fall. When the four phases of the dive were analyzed separately it was clear that HR was significantly lower during the free fall, than during the phases involving active swimming. When HR is less affected by the muscular work, as during free fall, apparently a more pronounced bradycardia can develop, likely resulting in oxygen conservation, which may lead to a less reduced SpO_2_. Passive free fall has been observed in deep diving seals, and is interpreted as a means to save energy during the descent by using the negative buoyancy to their advantage for prolonged gliding and thus minimizing active swimming (Williams et al., 2000).

During ascent, there was a minor decrease in HR in Phase 4, when the diver’s muscular efforts to reach the surface were reduced by positive buoyancy. The lower HR in Phase 3 compared to Phase 1, despite similar swimming efforts, could be due to the severe hypoxia during Phase 3. It has been shown previously in a study involving static apneas, that elite freedivers, but not non-divers, had a bi-phasic decrease in HR, and the authors ascribed the second drop in HR to the effects of their pronounced hypoxia (Lemaitre et al., 2008). We also interpret the remaining low HR during the initial 15 s of recovery to be a result of the prevailing hypoxia, before the resumed breathing and oxygen uptake has resulted in oxygen delivery to the tissues. These four phases are not evident in the shallower dives, during which the diver constantly swims.

The HR pattern found in our deep dives aligns with the previous recording of HR in one diver during a dive to 44 m (Schagatay, 2011), where it also appeared that the dive consists of different phases. Our present results contrast, however, to the description of HR responses in the study by Lemaitre et al. (2013) who measured ECG in deep dives of nine elite freedivers, diving to a depth of 70 ± 7 m, similar to the depth reached by our divers. They found that HR increased abruptly at 2/3 of the ascent, despite the positive buoyancy and less muscular effort, and concluded that diving HR is not directly affected by work intensity (Lemaitre et al., 2013). Based on our observation of a slight decrease in HR during the last phase, we find it more likely that the resulting diving HR is a net-effect of both stimuli where the opposite influences of the diving response (bradycardia) and exercise (tachycardia) on HR balance oxygen conservation with local energetic demands. In a study involving passive dives to 10 m in freedivers, Marabotti et al. (2009) showed that a reduction in both stroke volume and cardiac output accompanied the observed bradycardia, supporting the conclusion of an oxygen conserving diving response in humans. The average of 46% HR reduction observed in our study shows that the diving response in elite divers may be of similar magnitude as the 40% bradycardia observed in Steller sea lions (Hindle et al., 2010), while in gray seals average heart rate was shown to be reduced by nearly 90% (Thompson and Fedak, 1993).

Lemaitre et al. (2013) found a lowest HR of 23 bpm in one diver for a 10 s period, which is comparable to the lowest value of 28 bpm we observed. The HR reduction is influenced by the difference between ambient air and water temperature (Schagatay and Holm, 1996). In our study, there was a 4°C difference between ambient air and sea surface temperature, but a lower temperature at depth may have enhanced the bradycardia. Our data further suggests that there may not be an effect of depth beyond 20 m on the diving response, while comparing dives to 3 and 10 m in the study by Marabotti et al. (2008) revealed a larger HR reduction in the deeper dives. The resulting HR during apnea is affected by lung volumes in the upper range *via* stimulation of lung stretch receptors (Andersson and Schagatay, 1998), but we think this effect may cease after the relatively deep “shallower” dive of our study, when lung volume approaches residual due to lung compression.

### Limitations

The main limitation of this study is the small sample size, since only four divers were studied, which only allows preliminary conclusions to be made. However, the fact that all divers showed similar general responses may allow some observations concerning general events that we intend to study further in a larger group. The short periods with loss of data were handled by using an averaging method, which consequently showed continuous changes in HR and SpO_2_ in line with previous observations in the laboratory, which is promising for future underwater studies in freediving and other diving physiology. However, due to the possible influence of disturbances like involuntary breathing movements, equalizations, and hook breathing on the quality of the plethysmograms, further development is required to improve data quality. Another challenge is the reliability and precision of pulse oximetry when values drop below 70% SpO_2_, which is a problem in general but may be especially difficult to address when studying deep freediving, where direct comparisons to other methods may not be possible. Decreased oxygen saturation is central to studies on human hypoxia during freediving, and the new underwater pulse oximeter must therefore be further tested and validated both on land and underwater.

## Conclusion

Our study shows that monitoring SpO_2_ and HR with pulse oximetry is possible in an extreme underwater hyperbaric environment, which may be an important step for future tele-monitoring to enhance understanding of freediving physiology and diving safety. The observed patterns of SpO_2_ and HR were in line with previous observations done in the laboratory. Comparisons of our HR results to a few previous underwater studies also suggest that our recordings were successful. SpO_2_ may apparently be reduced to levels below 50% during ascent from deep dives, values considered incompatible with consciousness in non-divers, confirming an enhanced hypoxia tolerance in elite divers, but also suggests a considerable individual response. Our results support the hypothesis that effects of depth on gas exchange may enhance risk of hypoxic blackout during ascent. We further conclude that different energetic demands, relating to the four phases of deep freediving, may result in a balance between the opposing sympathetic exercise and the parasympathetic diving response stimuli, leading to an optimization of the diving bradycardia. The diving response also seems to be independent of depth beyond 20 m. With our results being based on only four divers with a total of eight dives, these findings need to be further investigated in a larger group. A next step could be to transfer data in real-time to the surface, which would not only allow future physiological studies but could also enhance freediving safety; a diver’s low SpO_2_ or high HR could call for attention and preparation of rescue measures at the surface.

## Data Availability Statement

The datasets presented in this article are not readily available as this conflicts with our ethics agreement with the participants. Requests to access the datasets should be directed to eric.mulder@miun.se.

## Ethics Statement

The studies involving human participants were reviewed and approved by Regional Committee for Medical and Health Research Ethics in Umeå, Sweden. The patients/participants provided their written informed consent to participate in this study.

## Author Contributions

EM contributed to the study design, planning and organization of field study tests and procedures, data collection, data analysis, and the manuscript writing. AS contributed with the original idea to build an underwater pulse oximeter, to construction and use of the underwater pulse oximeter, to data interpretation and analysis, and revising the manuscript. ES contributed with the original idea, planning and organization of field study tests and procedures, data analysis, and the manuscript writing. All authors contributed to the article and approved the submitted version.

### Conflict of Interest

The authors declare that the research was conducted in the absence of any commercial or financial relationships that could be construed as a potential conflict of interest.

## References

[ref1] Andersson J. Linér M. Fredsted A. Schagatay E. (2004). Cardiovascular and respiratory responses to apneas with and without face immersion in exercising humans. J. Appl. Physiol. 96, 1005–1010. 10.1152/japplphysiol.01057.2002, PMID: 14578373

[ref2] Andersson J. P. A. Linér M. H. Rünow E. Schagatay E. K. A. (2002). Diving response and arterial oxygen saturation during apnea and exercise in breath-hold divers. J. Appl. Physiol. 93, 882–886. 10.1152/japplphysiol.00863.2001, PMID: 12183481

[ref3] Andersson J. Schagatay E. (1998). Effects of lung volume and involuntary breathing movements on the human diving response. Eur. J. Appl. Physiol. 77, 19–24. 10.1007/s004210050294, PMID: 9459516

[ref4] Bakovic D. Valic Z. Eterovic D. Vukovic I. Obad A. Marinovic-Terzic I.. (2003). Spleen volume and blood flow response to repeated breath-hold apneas. J. Appl. Physiol. 95, 1460–1466. 10.1152/japplphysiol.00221.2003, PMID: 12819225

[ref5] Barker S. J. Shah N. K. (1997). The effects of motion on the performance of pulse oximeters in volunteers (revised publication). Anesthesiology 86, 101–108. 10.1097/00000542-199701000-00014, PMID: 9009945

[ref6] Craig A. B. (1961). Underwater swimming and loss of consciousness. JAMA 176, 255–258. 10.1001/jama.1961.03040170001001, PMID: 13696185

[ref7] Divers Alert Network (2017). A report on 2015 diving fatalities, injuries and incidents. Durham, NC: Divers Alert Network. 134. 29553634

[ref8] Fernández F. A. Rodríguez-Zamora L. Schagatay E. (2019). Hook breathing facilitates SaO_2_ recovery after deep dives in freedivers with slow recovery. Front. Physiol. 10:1076. 10.3389/fphys.2019.01076, PMID: 31543823 PMC6729099

[ref9] Ferretti G. (2001). Extreme human breath-hold diving. Eur. J. Appl. Physiol. 84, 254–271. 10.1007/s004210000377, PMID: 11374109

[ref10] Ferrigno M. Ferretti G. Ellis A. Warkander D. Costa M. Cerretelli P.. (1997). Cardiovascular changes during deep breath-hold dives in a pressure chamber. J. Appl. Physiol. 83, 1282–1290. 10.1152/jappl.1997.83.4.1282, PMID: 9338438

[ref11] Hindle A. G. Young B. L. Rosen D. A. S. Haulena M. Trites A. W. (2010). Dive response differs between shallow- and deep-diving Stellar sea lions (Eutmetopias jubatus). J. Exp. Mar. Biol. Ecol. 394, 141–148. 10.1016/j.jembe.2010.08.006

[ref12] International Association for the Development of Apnea (2021). World records. Available at: https://worldrecords.aidainternational.org/ (Accessed January 7, 2021).

[ref13] Kuch B. Koss B. Dujic Z. Buttazzo G. Sieber A. (2010). A novel wearable apnea dive computer for continuous plethysmographic monitoring of oxygen saturation and heart rate. Diving Hyperb. Med. 40, 34–40. PMID: 23111837

[ref14] Lanphier E. H. Rahn H. (1963). Alveolar gas exchange during breath-hold diving. J. Appl. Physiol. 18, 471–477. 10.1152/jappl.1963.18.3.471, PMID: 31083864

[ref15] Lemaitre F. Buchheit M. Joulia F. Frontanari P. Tourny-Chollet C. (2008). Static apnea effect on heart rate and its variability in elite breath-hold divers. Aviat. Space Environ. Med. 79, 99–104. 10.3357/ASEM.2142.2008, PMID: 18309906

[ref16] Lemaitre F. Lafay V. Taylor M. Costalat G. Gardette B. (2013). Electrocardiographic aspects of deep dives in elite breath-hold divers. Undersea Hyperb. Med. 40, 145–154. PMID: 23682546

[ref17] Lemaitre F. Polin D. Joulia F. Boutry A. Le Pessot D., Chollet., et al. (2007). Physiological responses to repeated apneas in underwater hockey players and controls. Undersea Hyperb. Med. 34, 407–414. PMID: 18251437

[ref18] Lin Y. C. Lally D. A. Moore T. O. Hong S. K. (1974). Physiological and conventional breath-hold breaking points. J. Appl. Physiol. 37, 291–296. 10.1152/jappl.1974.37.3.291, PMID: 4415069

[ref19] Lindholm P. Lundgren C. E. G. (2006). Alveolar gas composition before and after maximal breath-holds in competitive divers. Undersea Hyperb. Med. 33, 463–467. PMID: 17274316

[ref20] Lindholm P. Lundgren C. E. (2009). The physiology and pathophysiology of human breath-hold diving. J. Appl. Physiol. 106, 284–292. 10.1152/japplphysiol.90991.2008, PMID: 18974367

[ref21] Linér M. H. Ferrigno M. Lundgren C. E. G. (1993). Alveolar gas exchange during simulated breath-hold diving to 20 m. Undersea Hyperb. Med. 20, 27–38. PMID: 8471957

[ref22] Marabotti C. Belardinelli A. L’Abbate A. Scalzini A. Chiesa F. Cialoni D.. (2008). Cardiac function during breath-hold diving in humans: an echocardiographic study. Undersea Hyperb. Med. 35, 83–90. PMID: 18500072

[ref23] Marabotti C. Scalzini A. Cialoni D. Passera M. Ripoli A. L’Abbate A.. (2009). Effects of depth and chest volume on cardiac function during breath-hold diving. Eur. J. Appl. Physiol. 106, 683–689. 10.1007/s00421-009-1068-8, PMID: 19424715

[ref24] Marongiu E. Crisafulli A. Ghiani G. Olla S. Roberto S. Pinna M.. (2015). Cardiovascular responses during free-diving in the sea. Int. J. Sports Med. 36, 297–301. 10.1055/s-0034-1389969, PMID: 25429549

[ref25] Muth C. M. Radermacher P. Pittner A. Steinacker J. Schabana R. Hamich S.. (2003). Arterial blood gases during diving in elite apnea divers. Int. J. Sports Med. 24, 104–107. 10.1055/s-2003-38401, PMID: 12669255

[ref26] Örnhagen H. Schagatay E. Andersson J. Bergsten E. Gustafsson P. SandstrÖm S. (1998). “Mechanisms of ‘buccal pumping’ (‘lung packing’) and its pulmonary effects” in 24th Annual Scientific Meeting, European Underwater and Baromedical Society. August 12–15, Stockholm, Sweden, 80–83.

[ref27] Schagatay E. (2009). Predicting performance in competitive apnea diving. Part I: static apnoea. Diving Hyperb. Med. 39, 88–99. PMID: 22753202

[ref28] Schagatay E. (2011). Predicting performance in competitive apnea diving. Part III: deep diving. Diving Hyperb. Med. 41, 216–228. PMID: 22183699

[ref29] Schagatay E. Andersson J. (1998). Diving response and apneic time in humans. Undersea Hyperb. Med. 25, 13–19. PMID: 9566082

[ref30] Schagatay E. Holm B. (1996). Effects of water and ambient air temperatures on human diving bradycardia. Eur. J. Appl. Physiol. 73, 1–6. 10.1007/BF00262802, PMID: 8861662

[ref31] Stanek K. S. Guyton G. P. Hurford W. E. Park Y. S. Ahn D. W. Qvist J.. (1993). Continuous pulse oximetry in the breath-hold diving women of Korea and Japan. Undersea Hyperb. Med. 20, 297–307. PMID: 8286984

[ref32] Thompson D. Fedak M. A. (1993). Cardiac responses of grey seals during diving at sea. J. Exp. Biol. 311, 788–796. 10.1242/jeb.174.1.139 8440964

[ref33] Williams T. M. Davis R. W. Fuiman L. A. Francis J. Le Boeuf B. J. Horning M.. (2000). Sink or swim: strategies for cost- efficient diving by marine mammals. Science 288, 133–136. 10.1126/science.288.5463.133, PMID: 10753116

