# Corrigendum: Using Underwater Pulse Oximetry in Freediving to Extreme Depths to Study Risk of Hypoxic Blackout and Diving Response Phases

**DOI:** 10.3389/fphys.2021.700804

**Published:** 2021-07-19

**Authors:** Eric Mulder, Arne Sieber, Erika Schagatay

**Affiliations:** ^1^Environmental Physiology Group, Department of Health Sciences, Mid Sweden University, Östersund, Sweden; ^2^Swedish Winter Sports Research Centre, Mid Sweden University, Östersund, Sweden

**Keywords:** breath-hold diving, apnea, arterial oxygen saturation, syncope, heart rate, bradycardia, exercise, oxygen conservation

In the original article, we neglected to include the author, Arne Sieber. The corrected Author Contributions Statement appears below.

“EM contributed to the study design, planning and organization of field study tests and procedures, data collection, data analysis, and the manuscript writing. AS contributed with the original idea to build an underwater pulse oximeter, to construction and use of the underwater pulse oximeter, to data interpretation and analysis, and revising the manuscript. ES contributed with the original idea, planning and organization of field study tests and procedures, data analysis, and the manuscript writing. All authors contributed to the article and approved the submitted version.”

In the original article, the Funding Statement was incomplete. The new, correct Funding should be: “Funding was obtained through a donation from the Francis family in memory of their son/brother, who drowned from hypoxic blackout while snorkeling and holding his breath to dive underwater, and by a grant from the Swedish Research Council for Sport Science (CIF).”

The authors apologize for this error and state that this does not change the scientific conclusions of the article in any way. The original article has been updated.

